# Increased costs reduce reciprocal helping behaviour of humans in a virtual evacuation experiment

**DOI:** 10.1038/srep15896

**Published:** 2015-11-06

**Authors:** Nikolai W. F. Bode, Jordan Miller, Rick O’Gorman, Edward A. Codling

**Affiliations:** 1 Department of Engineering Mathematics, University of Bristol, Bristol, BS8 1UB, UK; 2 Department of Mathematical Sciences, University of Essex, Colchester, CO4 3SQ, UK; 3 Department of Psychology, University of Essex, Colchester, CO4 3SQ, UK

## Abstract

Altruistic behaviour is widespread and highly developed in humans and can also be found in some animal species. It has been suggested that altruistic tendencies can depend on costs, benefits and context. Here, we investigate the changes in the occurrence of helping behaviour in a computer-based experiment that simulates an evacuation from a building exploring the effect of varying the cost to help. Our findings illuminate a number of key mechanistic aspects of human decision-making about whether to help or not. In a novel situation where it is difficult to assess the risks associated with higher costs, we reproduce the finding that increasing costs reduce helping and find that the reduction in the frequency of helping behaviour is gradual rather than a sudden transition for a threshold cost level. Interestingly, younger and male participants were more likely to help. We provide potential explanations for this result relating to the nature of our experiment. Finally, we find no evidence that participants in our experiment plan ahead over two consecutive, inter-dependent helping opportunities when conducting cost-benefit trade-offs in spontaneous decisions. We discuss potential applications of our findings to research into decision-making during evacuations.

Helping behaviour is a defining feature of humans and is an extensively studied aspect of human behaviour^1^,^2^. In particular, altruism, helping others at a cost to the self, is widespread and highly developed in humans and can also be found in some animal species^3^,^4^,^5^. At first glance these behaviours are puzzling: why engage in costly actions that benefit others and are therefore likely to reduce the fitness of individuals? However, over the past decades a rich set of theories has been developed and tested to explain the nature and origins of altruism and helping in both animals and humans^6^,^7^,^8^,^9^,^10^,^11^.

These theories identify factors such as relatedness and familiarity as key predictors of altruistic behaviour. But a key challenge is to understand altruism directed to strangers, or individuals near enough to that status. Factors such as the apparent risks involved, whether one’s reputation is at stake^12^, the personal attachment to those in need^13^, and whether others in the vicinity could provide help instead^14^ have been identified as being relevant. More generally, it has been suggested that altruistic tendencies can depend on costs, benefits and context^5^. This concept is in line with evolutionary models of altruism, established with Hamilton’s Inclusive Fitness Theory^6^,^15^, that identify costs and benefits as key parameters for any helping behaviour.

In some cases, decisions on helping behaviour can be deliberated carefully over longer periods of time^1^,^2^ (e.g. decisions on volunteering^16^), but in other cases decisions have to be made in a relatively short time frame (e.g. assist an injured person during an evacuation). Here, we focus on the latter case, spontaneous decisions, and investigate the changes in the occurrence of helping behaviour depending on a key theoretical factor that is likely of relevance in real-world evacuation dilemmas: how costly it is to help another individual when there is uncertain but imminent risk. If individuals take account of different cost levels in their decisions, this is indicative of a trade-off between the motivation to help and perceived costs.

Previous work already provides evidence that increased costs can reduce the frequency or strength of helping behaviour^17^,^18^,^19^. We seek to increase our understanding of this phenomenon in three ways. First, we test whether a cost effect on helping can be reproduced in a situation where it is difficult to assess the risks associated with higher costs. In contrast to economic games with clear payoffs and costs^17^,^18^, in many real-life situations the risks and rewards are unquantifiable and the situation is only experienced once so that it is unlikely that prior experience can be used directly. Our experiment mimics such a situation. Second, provided there is a cost effect, we explicitly investigate how helping behaviour changes with increasing costs in a structured and carefully controlled manner. We test if the frequency of helping behaviour changes gradually or if there is a sudden transition for a threshold cost level. This could provide fundamental insights into the mechanisms of the trade-off between the motivation to help and perceived costs. Third, we test whether people plan ahead over two consecutive and inter-dependent helping opportunities when conducting such trade-offs in spontaneous decisions.

Our experiment simulated an evacuation from a building. We chose this particular context, because evacuations are an easy to understand scenario in which opportunities to help others, under uncertain risk levels, are frequently presented to people^20^. Furthermore, understanding helping behaviour in evacuations is important and could have practical applications. We discuss the extent to which our work is helpful in this sense in more detail below.

To be able to control the experiment in a comprehensive and safe way, we tested participants’ behaviour in a virtual environment, the simulation of which was based on previously established methodology^21^,^22^,^23^. Such computer-based paradigms on human decision-making are widely and successfully used in a range of contexts^24^,^25^,^26^, including evacuations^21^,^22^,^23^,^27^. Particularly relevant to the present study is the work by Drury and co-workers^27^ who found, using experiments on helping behaviour in a virtual environment, support for their theory that a collective social identity emerges from the shared experience of an emergency and promotes helping behaviour towards strangers. Drury *et al.*’s work suggests that a virtual environment is a promising tool for studying helping behaviour.

The findings from research using virtual environments to study human behaviour must be interpreted carefully. As for many experimental abstractions of real-life scenarios, it is not clear to what extent behaviour in experiments matches behaviour in real life^28^. With this in mind, we used our experimental paradigm not to establish absolute real-world levels of helping behaviour, but to test relative changes in helping behaviour to gain novel insights into human decision-making.

## Experiment

**Figure 1:**
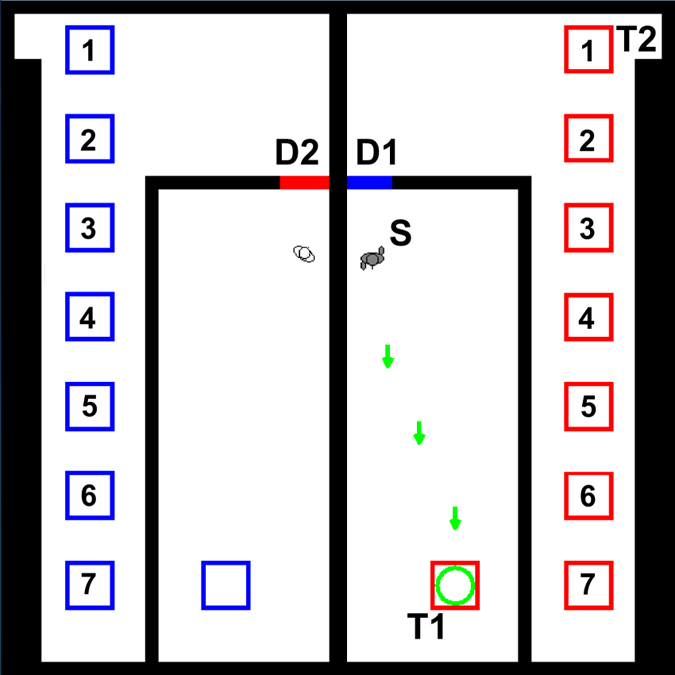
Layout of virtual environment. We show the start of the training phase with the computer-controlled pedestrian (shown in white) and the participant-controlled pedestrian (shown in grey) located in the central rooms of the left and right hand halves of the building, respectively. The participant-controlled pedestrian is at the starting position, S. Moving pedestrians have animated legs that are not visible when pedestrians are stationary (compare participant-controlled and computer-controlled pedestrians). Green arrows indicate the direction towards the first target, T1 that coincides with a coloured square that can be used for opening door D2 (arrows disappear when T1 is reached). The computer-controlled pedestrian can open door D1 by standing on one of the blue squares in the left hand half of the building. The final exit from the building for the participant is labelled ‘T2’. We show all 7 possible locations for the coloured square in the corridor (labelled 1–7). In the experiment, only one square at one of these locations was shown and we showed squares at the same location in both halves of the building. For the computer-controlled pedestrian to escape, the human participant must stand still on the red square in the corridor on their side of the building. By varying the location of this red square we can test how the perceived relative cost affects helping behaviour.

Our experiment simulated an evacuation of two pedestrians from a building, presenting a top-down view of a virtual environment (Fig. 1). One of the simulated pedestrians was controlled by the participant, the other was controlled by the computer program (although participants were not told that the other pedestrian was computer-controlled). To be able to exit the building, the two pedestrians had to open exit doors for each other. We tested whether participants helped the other pedestrian by opening exit doors and we varied the amount of time participants had to invest to do so. The experiment comprised a training phase and a test phase. Participants were only allowed to complete each of these phases once. In the training phase, participants were taught how to control the movement of their pedestrian inside a virtual building. The simulated evacuation inside this virtual environment took place in the test phase. We next describe the virtual environment, then the training phase and finally the test phase and experimental treatments.

The virtual environment was a simplified representation of a building. Contextual information and instructions to participants were provided via popup windows, triggered sequentially as participants followed instructions. Inside the virtual building were two simulated pedestrians. Participants controlled the designated pedestrian (indicated by a colour-code) using a mouse, while the other pedestrian was controlled directly by the computer program, as noted earlier. The virtual building was symmetrical with two halves separated by a wall. Each half contained within it a central room and a remaining corridor-like peripheral area, with each central room connected by a door to the corridor. By default, both connecting doors in each half were closed. The pedestrians could open the door in the *opposite* half of the building by standing within one simulated pedestrian diameter of the centre of either of two coloured squares located in their *own* half of the building. Doors only remained open for as long as pedestrians remained close enough to the centre of a coloured square and closed immediately when pedestrians moved further away. In each half of the building, one of the two coloured squares was inside the central room and the other was in the corridor. All coloured squares were visible throughout the experiment. Each corridor also contained a target exit from the building (labelled ‘T2’ in Fig. 1).

In the training phase, participants were taken through a series of steps, guided by instructions, designed to teach them how to interact with the virtual environment. We outline the content of instructions here and present the precise wording of the instructions in the methods section, as well as in supplementary video S1, which shows a complete experimental run. At the start of the training phase of the experiment, the participant-controlled and the computer-controlled pedestrian were situated in the central rooms of the opposite halves of the building (see Fig. 1). Participants were then instructed to move from the starting position, ‘S’, to the first target, ‘T1’ which took the form of a coloured square located within the same room. Upon completion of this step, participants opened the door in the opposite room, as explained earlier. As a result, participants were taught how movement onto the (red and blue) coloured squares would open the doors. In addition, upon reaching target ‘T1’, participants were told that they would need help to exit the building, and were instructed to return to ‘S’. Participants were not informed explicitly that the other pedestrian (computer-controlled) also required help to exit the building. We therefore tested helping behaviour in a situation where helping opportunities arise, but are not introduced explicitly, as is likely to be the case in many real-world situations. Throughout these tasks, the computer-controlled pedestrian remained unmoved at its starting position.

Once participants had returned to the starting position, the test phase of the experiment began, with a message advising them that they now had to quickly leave the building because there was an emergency (see Methods and supplementary video S1 for full details). No information on the amount of time available was provided. Following this message, the computer-controlled pedestrian began to walk directly towards the coloured square in its central room, which opened the door for the participant (i.e. door ‘D1’ in Fig. 1). Then the computer-controlled pedestrian waited until the participant-controlled pedestrian left their central room. Thus, the participant-controlled pedestrian was always the first to exit a room. Once this occurred, the computer-controlled pedestrian walked towards the door that led to the corridor (i.e. ‘D2’). By walking towards the door, the computer-controlled pedestrian provided a cue for participants indicating that they could help. Participants were not informed that the computer-controlled pedestrian always followed this sequence of actions. If the participant opened the door for the computer-controlled pedestrian by moving to the second square in the corridor, then the computer-controlled pedestrian left the room and exited the building.

During the test phase, the coloured square in the participants’ corridor was located at one of seven possible options and this variation in location presented the experimental treatment. Our virtual environment was symmetrical and the location of the coloured squares for the computer-controlled pedestrian were adjusted in the same way. This treatment altered the length of the detour participants had to make in order to open the door for the computer-controlled pedestrian from the corridor (see Fig. 1). Making longer detours in the virtual environment took more time and required additional effort (in terms of clicking with the computer mouse). In this way, the different locations for the coloured square in the corridor imposed different costs for helping. We did not explain the costs for helping or the different cost levels to participants. Our experiment introduced an uncertain risk level by suggesting that participants had to evacuate quickly without giving an actual time limit and we were interested in how increased times required for helping affected participants’ decision to help under this uncertain risk. For simplicity, we quantified costs in terms of the vertical distance from the on-screen position of the exit from the building (‘T2’). Square location 1 in Fig. 1 had associated cost 0 and subsequent square locations had costs that were incremented by constant amounts from this baseline. In supplementary videos S1 and S2 we show one complete experimental run for cost level 0 (square location 1 in Fig. 1) and cost level 6 (square location 7 in Fig. 1), respectively.

To ensure that we had a balanced distribution of participants across the seven cost levels (i.e. similar numbers of participants experiencing each of the cost levels), we used the following procedure. Each participant was assigned a unique integer number. This number was incremented by 1 between consecutive participants (e.g. first participant has number 1, second participant has number 2 and so on). The cost level a given participant experienced was determined by modulo 7 of her unique participant number (e.g. participant number 7 implies cost level 0). As participants were not allowed to watch others and only completed the task once, they were not aware of this procedure.

Our computer program recorded the on-screen location of both pedestrians in the virtual environment for each update step of the underlying computer simulation. Details on the computer simulation can be found in the methods section.

### Data collection

As part of a ‘Live Science’ residency at the Science Museum, London, we invited museum visitors to take part in our experiment. Between the 2^nd^ and the 11^th^ of September 2014, a total of 632 people took part in the experiment. We ensured that people only participated once and that people who had not yet participated did not watch others before taking part. We had to remove data from a total of 62 participants from our analysis. This was because participants terminated the program before data was saved (13 participants), because participants did not report their age or gender (40 participants), or because participants were too young to take part without the help of their parents (9 participants younger than 7 years – age was recorded anonymously via the computer program and only accessed during analysis). Therefore, our analysis is based on the data from 570 participants who were split across the six cost levels as follows: 0 (81), 1 (86), 2 (80), 3 (83), 4 (79), 5 (79), 6 (82). The gender split was fairly even with 290 men (50.9%) and 280 women (49.1%). The median age across participants was 23 years (mean 26.44 years); ranging from 7 years old to 70.

### Research questions

Our experiment presented participants with two opportunities for offering to help. The first opportunity occurred when participants were still inside the central room and could choose to help by moving towards the coloured square to open door ‘D2’. However, this did not alter the behaviour of the computer-controlled pedestrian, which always travelled to the coloured square that opened door D1 and would stay there until the participant had exited their room. Once participants exited their central room through door ‘D1’, the computer-controlled pedestrian then left the coloured square in their room and attempted to exit (but was unable to do so as door ‘D2’ was closed). Once in the corridor, the second opportunity for the participant to help the computer-controlled pedestrian occurred. Participants could choose to move onto the coloured square in the corridor to open door ‘D2’ or instead walk straight to the exit from the building (‘T2’). Videos illustrating these events occurring during the game are available as supplementary material (Supplementary videos S1 and S2). We investigate the decision of participants at both of these helping opportunities.

We adopt the following notations and definitions (also summarised in Table 1). Event A denotes whether participants offered to help at the first opportunity by opening the door for the computer-controlled pedestrian whilst still inside the central room (i.e. A occurred if participants tried to help from inside the central room). Event B denotes whether participants helped at the second opportunity and occurred if the computer-controlled pedestrian left the central room, as it could then exit the building without any further help. To further explore participants’ behaviour in the corridor, we defined event C to be the case when participants opened the door for the computer-controlled pedestrian from the corridor. If event C occurred (i.e. participants opened the door from the corridor), this did not necessarily imply that the computer-controlled pedestrian exited the central room (event B), as the door could close again before this had happened. Throughout, we denote the proportion of participants for whom events A, B and C occurred by P(A), P(B) and P(C) (e.g. P(B) is the proportion of participants who helped at the second opportunity).

At the first helping opportunity (also the start of the test phase), participants had no prior experience of the behaviour of the other pedestrian (computer-controlled) but possessed all information required to make a judgement of detours required in order to help inside and outside of the central room. Different ways in which participants might assess their situation lead to separate, mutually exclusive hypotheses for how cost levels affect the probability of event A (first helping opportunity) to occur, P(A).

*H1a: cost levels do not affect P(A).* This situation arises either if participants do not take the location of the coloured square outside of the central room into account or if participants generally disregard cost levels. The latter scenario is plausible, as the only real cost to participants is time loss. Thus, as an example, cultural norms to help, exacerbated by the knowledge that data is collected, may prompt participants to help regardless of the virtual-environment risks.

*H1b & H1c: cost levels affect P(A).* This requires participants to take the situation in the corridor into consideration, specifically the location of the square to open the computer-controlled pedestrian’s door, and to make an overall assessment of how to minimise their investment in terms of how far they have to walk, for example. The higher the cost level, the larger the required detour outside of the central room that is needed in order to help. If participants assume that the other pedestrian will reciprocate their help, higher cost levels could make helping inside the central room more attractive, either gradually (H1b; e.g. a linear response to the apparent cost) or suddenly, once a threshold cost level is reached (H1c; e.g. when the length of the detour outside the central room exceeds the detour inside the central room, participants may be much more likely to attempt helping inside the central room).

Analogously to hypotheses H1a-c, we can formulate hypotheses for P(B), the probability for participants to help at the second opportunity, after they have exited the central room. Here, the question is whether participants reciprocate the help they invariably received whilst inside the central room.

*H2a: cost levels do not affect P(B):* This would arise if participants completely disregard cost levels during the second helping opportunity.

Hypotheses H2b and H2c predict cost level effects on P(B) in a similar way to hypotheses H1b (e.g. linear response to cost) and H1c (threshold response to cost) above. In particular, hypothesis H2c predicts that participants’ helping behaviour changes sharply at a threshold cost. For example, it may be that participants are only willing to make a detour of a similar or smaller size than the detour made by the computer-controlled pedestrian.

The main aim of our experiment was to explore the effect of different cost levels on the probability for participants to help. In addition, we considered a number of covariates that could help to explain how participants responded to the experimental tasks. Specifically, we investigated whether age, gender and the lengths of the two time intervals participants needed to complete two movement stages in the training phase (move from start position to T1 and return from T1 to start position) had consistent effects on helping behaviour. We included covariates on gender and age for exploratory purposes and we included covariates on training times as one approach to account for potential differences in participants’ familiarity with interactive virtual environments. Table 1 provides an overview of all summary statistics and covariates used in our analysis.

In summary, we conducted a computer-based experiment to test how different levels of required investment affect helping behaviour of humans under uncertain risks and without an explicit payoff. We were particularly interested to determine to what extent helping behaviour may be governed by considerations such as a minimisation of the distance covered and forward planning. In addition, we explore whether the probability of helping changes gradually with the apparent cost or if this relationship is governed by a threshold or step-change in behaviour as the cost increases.

## Results

### Initial observations

**Figure 2:**
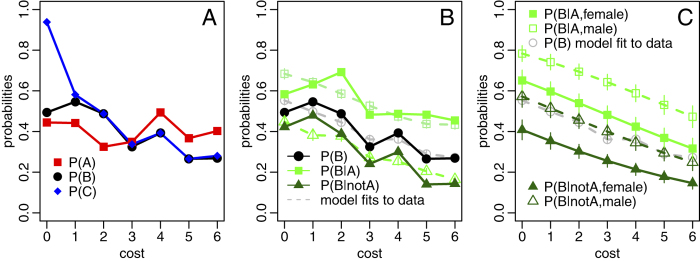
Probabilities for events occurring estimated from the observed data. Panel (**A**) shows probabilities estimated from the experimental data (event A – participant opens the door for the other pedestrian from the central room, event B – computer-controlled pedestrian gets rescued, event C – participant opens the door for the other pedestrian from the corridor). Panel (**B**) shows P(B) conditional on the occurrence of event A. Dashed lines indicate the fit of the model presented in Table 3 to the observed data. Panel (**C**) shows probabilities for events occurring predicted from model fits to the data. We show the fit of the model to the observed data and the predicted values for P(B) conditional on the occurrence of event A and the gender of participants. This highlights the effect behaviour inside the central room and participant gender have on P(B). For clarity of illustration, the effect of age is not shown here. As an indication, 11 years of age difference produce the same effect size as gender (see Table 3). In predictions from the statistical model, we set the age to the median value (23 years) and the explanatory variables for training data to their mean value. See text and Table 3 for descriptions of the statistical model. Error bars show standard errors estimated from model fits.

We first computed the fraction of participants who helped at the second opportunity, P(B), for the 7 different cost levels. Figure 2A shows that P(B) appeared to decrease for increasing levels of the cost. No such trend was immediately visible for P(A), the probability of participants to attempt helping whilst still inside the central room (Fig. 2A). Averaged across cost levels, we found P(A) = 0.40 ± 0.06 (mean ± s.d.). In contrast, P(C), the probability of participants to move onto the coloured square outside the central room, showed a strong decrease for increasing cost levels (Fig. 2A).

Interestingly, P(B) and P(C) did not always take the same values, in particular for cost level 0. This suggests that some participants walked onto the coloured square in the corridor (C occurs), but did not stay there for long enough to allow the computer-controlled pedestrian to exit (B occurs). Figure 1 shows that for cost level 0, the coloured square is on the way to the final exit. It is therefore very difficult for participants to avoid walking onto the square under this experimental condition, whether they intended to help or not. For higher cost values, P(B) and P(C) took very similar values suggesting that only very few participants made a large detour to walk onto the coloured square and did not make sure they stayed there for long enough.

Figure 2B shows the proportion of participants who helped given that they had already tried to help whilst still inside the central room, P(B|A). For all cost values, P(B|A) was noticeably higher than P(B), which suggests that people who immediately tried to help were in general more likely to help on the second occasion they had the opportunity to do so (in the corridor). This figure provides a strong motivation to investigate whether participant behaviour inside the central room predicts the final outcome of the evacuation task.

To explore consistent trends in participants’ familiarity with virtual environments, such as ours, we conducted a preliminary analysis (see methods) on the time taken by participants to complete the two movement phases during training (move from start position to T1 and return from T1 to start position). This showed that older people and female participants took longer, on average. To account for these differences between genders and ages and to test to what extent training times affected subsequent behaviour, we therefore additionally used these training times as covariates in our subsequent analysis.

### No cost effect at first helping opportunity

We fit a generalised linear model (GLM) to our data to investigate the effect of cost levels on the proportion of participants who attempted to help at the first opportunity, P(A) (hypotheses H1a-c). We also investigated the effect of age, gender and training times in this model. Table 2 shows the results for the statistical model fit. As already indicated by Fig. 2A, we found no effect of cost levels on the probability of participants to help at the first opportunity, P(A) (p = 0.55, Table 2). We can therefore reject the hypotheses which suggested that such an effect would occur (H1b and H1c). There is a clear indication in the data that lower speeds for the second movement phase of training relate to reduced P(A) (p = 1.14 × 10^−2^, Table 2). However, differences in training did not suffice to explain apparent differences in behaviour related to gender and age: at the first helping opportunity men and younger participants were more likely to help (both p < 0.05, Table 2).

We also examined whether cost levels, instead of affecting P(A), could alternatively affect the length of time participants waited before exiting the central room. For example, participants who tried to help under higher cost levels may have waited longer for a reaction from the computer-controlled agent. However, we found no cost effect on the time participants spent inside the central room (Likelihood ratio test: Χ^2^(1) = 0.05, p = 0.82; waiting time was log-transformed to meet model assumptions).

### Cost levels affect helping at second opportunity

We next investigated cost-level effects on the probability for participants to help at the second opportunity, P(B) (hypotheses H2a-c) in a similar manner to our examination of P(A). Based on our initial observation from Fig. 2B, that participants who helped at the first opportunity were more likely to help at the second opportunity, we additionally included a binary explanatory variable for attempting to help at the first opportunity (event A) in our statistical model. Our statistical analysis confirmed that increasing cost levels resulted in a reduction of the probability to help at the second opportunity, P(B), and that this effect was unlikely to have arisen by chance (p = 1.60 × 10^−6^, Table 3). In the same way as for the probability to help at the first opportunity, P(A), we also found a clear indication that gender and age had an effect on P(B) in that female and older participants were less likely to help (both p ≪ 0.05, Table 3). Differences in participants’ training times did not help to explain the data (both p > 0.05, see Table 3), but the occurrence of event A did (if A occurred P(B) increased, p = 2.52 × 10^−7^, Table 3). Taken together, these results suggest that the decision to help at the second opportunity in participants was influenced by the associated cost, by personal characteristics (gender, age) and that participants’ behaviour inside the central room provided a substantial indication for their later behaviour in the corridor. Figure 2B shows that the statistical model provides a reasonable fit to the observed data. In addition, model predictions illustrate the considerable effect that behaviour inside the central room has, suggesting that regardless of the cost level, participants who try to help at the first opportunity (A occurs) are at least 1.5 times more likely to help at the second opportunity compared to participants who do not try to help immediately (Fig. 2C). Although not as strong, the effect of gender on the probability to help at the second opportunity, P(B), is also considerable (Fig. 2C). We also found that if we only considered data from participants who helped in the first place, there was still a cost level effect on the probability to help at the second opportunity, P(B|A) (Table 4).

Finally, we tested whether the effect of cost levels on the probability to help at the second opportunity, P(B), was a gradual response (hypothesis H2b), or whether there was a threshold cost level, beyond which participants’ helping behaviour changed sharply (hypothesis H2c). Figure 2A shows that P(B) takes values approximately within the interval [0.27,0.55] and it is not immediately obvious that there is a sudden change in P(B) as the cost level increases. To explore this intuition quantitatively, we proposed a number of models for P(B) that captured the contrasting hypotheses and computed the maximum likelihood fits for these models to our experimental data. We then used the Akaike Information Criterion (AIC) to determine which model best explains the data (AIC = 2k−2log(L), where k is the number of model parameters and L the maximum likelihood). Specifically, we considered models for linear, exponential, logistic and adjusted logistic decay of P(B). The first two models assume a gradual change in P(B) and the latter two models can capture a sharp transition in P(B). All model details are given in the methods section below. We found that the AIC values for the four different models do not differ substantially (AIC values for linear decay: 748.35; exponential decay: 748.85; logistic decay: 748.36; adjusted logistic decay: 751.72). We suggest that from our data it is not possible to determine with confidence how P(B) decays with increasing cost levels. However, parsimony and the relatively small range of P(B) values would suggest a gradual change in P(B) (hypothesis H2b) instead of a threshold response (hypothesis H2c). Conducting the same analysis separately for P(B|A) and P(B|notA) (i.e. the proportion of participants who helped at the second, but not at the first opportunity) yielded similar results and suggested that the adjusted logistic model consistently provided the worst fit to the data (see methods).

## Discussion

We have conducted an experiment in a virtual environment with over 500 participants to investigate helping behaviour in humans. The main focus of our experiment was to investigate how varying cost levels affect helping behaviour and whether there is evidence for forward-planning across two consecutive helping opportunities in a novel context that was inspired by real-life evacuation scenarios. In this way, we do not directly address the evolutionary origins of altruism or why people show helping behaviour, a topic which is subject to a lively debate^3^,^4^,^12^,^13^,^14^,^29^. Instead, we explore what helping behaviour depends on and we suggest that this will ultimately be useful in addressing questions on the origins of altruism.

Previous work has shown that the motivation to help may be traded-off against associated costs^17^,^18^,^19^,^30^. We contribute to the understanding of how costs affect helping behaviour by reproducing this finding in a scenario where the risks and rewards are unquantifiable and where it is unlikely that prior experience can be used directly. One possible interpretation for this result is that even in a novel and uncertain scenario, people estimate risks in a consistent way, on average. For our experiment, this would suggest that participants have a consistent estimate for the risk associated with taking longer to evacuate and they balance this risk against their motivation to help. Such a proposition is plausible, as humans have developed decision-making mechanisms for situations where relevant information is missing^31^. At this point it should also be noted that the relationship between interaction partners is likely to be important in this context. We tested interactions between strangers and found that increased costs reduced helping behaviour. However, an experiment based on questionnaires has shown that for interactions with kin, the willingness to help increases with increasing costs, but that it decreases for interactions with acquaintances or even friends^19^.

To our knowledge, the nature of the transition from high to low occurrence of helping behaviour with changing cost levels has to date been largely unexplored. Previous work has typically only considered very few cost levels^19^,^30^ or the strength of altruistic contributions rather than the occurrence of helping behaviour per se^18^. While it was difficult to establish exactly whether this transition from higher to lower helping frequencies was gradual or occurred for a threshold cost level, we suggest that based on our data a gradual change is more likely. Studying the nature of this transition is particularly useful in our opinion. In many contexts, humans use simple heuristics based on thresholds to make decisions^31^,^32^ and for our experiment it could have been reasonable to suggest that in the second stage participants only invest as much as the computer-controlled pedestrian had invested in the first stage, for example. Our findings do not clearly support such a threshold-based decision-making process, regardless of potential differences in motivation between participants as expressed at the first helping opportunity. This makes it less obvious to determine what simple heuristics humans could use in decisions on spontaneous helping behaviour. Addressing this issue in greater depth requires further tests across different contexts and using a wider range of cost levels. Such additional work could also address the limitation of our present study, which did not provide a clear-cut distinction between a gradual and a threshold change in helping behaviour with increasing costs.

An additional finding from our study was that participants did not appear to take likely costs to help at the second opportunity into account in determining whether to help at the first opportunity. In our experiment, the scenario presented participants with two connected helping opportunities. A priori, participants did not know what the other (computer-controlled) pedestrian would do. If participants had wanted to minimise the distance they had to walk across the entire evacuation, but still wanted to evacuate both pedestrians, we would have expected a cost effect on P(A), the probability to help at the first opportunity. If participants simply wanted to minimise their evacuation time, we would have expected P(B), the probability to help at the second opportunity, to be close to zero. Therefore, if participants did take costs at the second helping opportunity into consideration at the start of the evacuation, it is not immediately obvious how they did this. There are different possible explanations for this finding. Our cost levels only indirectly affected the investment necessary to help at the first opportunity. It may be that decision-making in a hurry relies on simple heuristics^31^ that may not include forward-planning and may not take indirect costs into consideration. Alternatively, participants may have simply not made the connection between the first and the second helping opportunity. These potential explanations could be tested in extensions of our experiment. For example, cost levels could be adjusted separately for both helping opportunities and the connection between helping opportunities could be explained to participants. Furthermore, different cost levels could be presented in the two halves of the virtual environment (i.e. coloured squares in the corridors are not both the same distance from the exit). In this way the strategy that minimises the combined cost for both players could be altered. This would facilitate further insights into the relative importance of reciprocal helping and overall cost reduction. What is clear, is that participants’ behaviour inside the central room helped to predict their subsequent behaviour. Participants who offered help in the first place were more likely to help at the second opportunity. This could be indicative of a split between people with stronger or weaker altruistic tendencies^12^. However, cost levels still affected the subsequent decision to help for participants who helped in the first place. Therefore, we find no evidence for a fundamental difference in decision-making mechanisms between participants who tried to help immediately and those who did not.

One inherent feature of our experimental setup is that we did not directly explain the costs present to participants. Therefore, we cannot be sure participants consciously traded off detour lengths against their motivation to help. To some extent this is deliberate, as we want to approximate real-life situations. However, this opens up the possibility for alternative explanations for our findings. For example, participants may have simply found it more difficult to spot the second helping opportunity, the further the coloured square was away from their exit point. We suggest that such alternative explanations are possible, but unlikely for a number of reasons. First, both squares were mentioned in the instructions (see methods). Second, for higher cost levels the coloured squares in the corridor were located in close proximity to the coloured square inside the central room, thus making it easier to spot the location of both coloured squares (see Fig. 1). This would suggest that the second helping opportunity should be the most difficult to spot for intermediate cost levels. Finally, this alternative explanation would still suggest that participants traded off their motivation to help against the time investment required to spot the second helping opportunity. Tracking the gaze direction of participants^33^ during our experiment would help to determine if participants spotted the second helping opportunity.

We found a consistent effect of gender and age on the probability to help. Older participants and female participants were on average less likely to help. On the one hand, previous work has suggested that there could be fundamental differences in male and female helping and risk-taking behaviour. For example, it has been suggested that higher generosity in men (when observed by women) may have evolved as a mating signal^34^. In addition, helping in our experiment involves taking a risk and previous research has shown that risk-taking levels are higher in men than in women across a number of contexts^35^, which may also be linked to sexual selection pressures^36^. On the other hand, both gender and age may be linked to previous experience of computer games with tasks and controls similar to our virtual environment^37^. In addition, based on previous experience of playing computer games, possibly related to age, there may be fundamental differences in the extent to which participants identify with the pedestrian they control which in turn is likely to affect helping behaviour^27^. We included participants’ performance during training into our statistical analysis and this may account for some of these aspects, but there is no guarantee that we have covered all such effects. Based on this experiment alone, we therefore suggest that while the age and gender effects we found are interesting and could be investigated further, they should only be considered directly within the context of our virtual environment.

Many extensions to our experiment are possible. Perhaps the most obvious refinement would be to let humans control both pedestrians in the experiment. This might change the extent to which individuals plan ahead and consider both helping opportunities at the start of the evacuation. Other important avenues for future work could be to repeat our experiment in more immersive virtual environments^27^,^38^ or in a real, physical environment with human participants to test the extent to which our findings depend on the specific experimental setup.

The context of our experiment, an evacuation from a building, suggests one potential application of our findings. Planning for such emergencies is essentially aimed at ensuring that all evacuees have enough time to reach safety, taking into account how long it takes to respond to an alarm and to move to safety^39^. Full-scale evacuation drills are expensive and potentially dangerous and as a result planners have increasingly turned to computer simulation models to explore the evacuation times for building, event venue and vehicle designs^40^. Some of these simulation models incorporate helping behaviour^41^. While the results of our computer-based experiment should not be interpreted and applied directly to real life human behaviour, we suggest that they could present an empirically founded intuition and starting point for how to implement helping behaviour algorithmically in evacuation simulations, as well as a starting point for more realistic (non-computer based) empirical studies.

## Methods

Our experiment was approved by the Ethics Committee of the University of Essex and conducted in accordance with the approved guidelines. We obtained informed consent to take part in the experiment from all participants.

### Virtual environment simulation

Pedestrian movement in the virtual environment was simulated using established methodology^21^,^22^,^23^. Pedestrians moved in continuous two-dimensional space. Preferred movement directions were indicated via mouse clicks (human-controlled pedestrian) or via pre-defined waypoints (computer-controlled pedestrian). The environment (e.g. walls) was encoded in a discrete floor field^42^ and interactions between pedestrians and the environment (e.g. avoiding walls) were modelled via forces acting on point masses, following previous theoretical work^43^. Pedestrian movement was simulated in fixed simulation update steps that corresponded to about 5 ms of real time. We set the simulation parameters to values that guaranteed smooth pedestrian movements at a speed that ensured participants had enough time to observe and control movements in the virtual environment (see refs 21, 22, 23 for details).

### Wording of instructions given to participants

Below, we detail the wording of the instruction messages displayed during the experiment. The simulation was paused while instructions were displayed.

The introductory screen and instructions were follows: *“Hello, in this game you are playing a person in a building. There is one more person in the building. You can move your person by clicking with the mouse where you want it to go. To start with, follow the green arrows on the floor to your first target—a green circle. Your person is shown in grey. When you are ready, press the START button above*”. After starting the game, the message *“Move to the green circle”* was displayed in a panel underneath the simulated environment. At this point participants who struggled to move their pedestrian were given the additional verbal instruction: *“You are the grey person here, and when you click somewhere, this is where you are going to move*”.

Upon reaching the first target, participants saw an instruction on how to open doors for 15 seconds: *“Well done! When you are inside one of the TWO red SQUARES, the red door opens. The blue door only opens when the person in the next room is inside a blue SQUARE. So you need help to exit the room!”* While this message was displayed, the doors were flashing to highlight their location. Subsequently, participants received further instructions that were displayed for 8 seconds: *“Head back to the position where you started the game. Before you go, try opening and closing the red door a few times. You need to practice your clicking skills!”* After these instructions had disappeared, a green circle appeared at the starting position and the message underneath the virtual environment display read: *“Move to the green circle when ready”*.

Reaching the green circle at the starting position triggered the evacuation phase that was initiated with the message (shown for 9 seconds): *“There has been an Accident. Evacuate the building as quick as possible. There is not much time for you to get out. Go to the NEW green circle. YOU must survive!”* This message was shown underneath the image of a commonly used emergency exit sign to highlight the urgency and to add context (see supplementary videos S1 and S2). The green circle was now displayed at location T2 (Fig. 1), indicating the new target location. When the simulation had re-started, participants could see the message, *“Exit the room (green circle)”,* in the panel underneath the simulated environment.

### Movement time in training affected by gender and age

To test if gender and age affected the length of the two movement phases during training (move from start position to T1 and return from T1 to start position), we fit linear regression models to the data with log-transformed training times as the response and age (continuous) and gender (categorical) as explanatory variables (models included an intercept). We report the results from single parameter tests. All statistical analysis was conducted in the R programming environment, version 3.0.1^44^. Normality and independence assumptions of the statistical models were verified to hold by assessing residual plots.

*Training 1.* Movement time from the start position to T1. With increasing age, this time increases (0.02 ± 2.50 × 10^−3^ (estimate, mean ± s.e.), 6.37 (Z value), p = 3.93 × 10^−10^). Differences in gender had no effect on this time (Gender (male): −0.06 ± 0.06 (estimate, mean ± s.e.), −0.96 (Z value), p = 0.34).

*Training 2.* Movement time from T1 back to start positon. As above, with increasing age, this time interval increases (0.01 ± 1.93 × 10^−3^ (estimate, mean ± s.e.), 3.73 (Z value), p = 2.15 × 10^−4^). For this time interval, we found evidence for a difference between genders (Gender (male): −0.13 ± 0.05 (estimate, mean ± s.e.), −2.70 (Z value), p = 7.09 × 10^−3^).

### Testing hypotheses H1a and H2a

Hypotheses H1a and H2a suggest that cost levels do not affect the proportion of participants who offer help at the first and second opportunity, respectively. To test for an effect of cost levels, as well as individual characteristics (age, gender), behaviour in training (Training 1, Training 2) on the probability for participants to help at the two different opportunities, we fit generalised linear models (GLMs) to the experimental data. The GLMs we fit to our data had the occurrence of event A or event B as the response variable (taking values 1 or 0 if helping did or did not occur, respectively) and had binomial error structure. All GLMs included an intercept and the explanatory variables we used are listed in Tables 2 and 3. We report all p-values for single parameter tests (parameter value equals zero under the null hypothesis). Independence assumptions of the statistical models were verified to hold by assessing residual plots.

### Testing hypotheses H2b and H2c

We propose models for the proportion of participants who help at the second opportunity, P(B), that capture a gradual change (as suggested by hypothesis H2b) or a sharp change for a threshold cost level (as suggested by hypothesis H2c). As we are only interested in the nature of the cost level effect on P(B), we disregard other explanatory variables (e.g. gender, age,…) in this analysis under the assumption that the distribution of participants with different characteristics across cost levels is approximately balanced (if this was not the case, it would be visible in the model fit to the data in Fig. 2B). Using the model expression for P(B), we can fit the models to the experimental data, maximising their likelihood. This was achieved in the R programming environment, version 3.0.1, by minimising the negative log likelihood using the ‘nlm’ function^44^. We report both the start values used in the minimisation procedure and the fitted values for all model parameters. AIC scores and therefore maximum likelihoods are given in the main text. The models we considered are listed below. In the following, the variable x always indicates the cost level and in computing likelihoods from the model expression for P(B), we set P(notB) = 1—P(B).

*Linear decay:* P(B) = a + bx, where a and b are model parameters. The start and fitted values for (a,b) are given by (0.1,−0.01) and (0.54,−0.05), respectively. This model captures a gradual decay in P(B).

*Exponential decay:* P(B) = exp(a + bx), where ‘exp’ is the natural exponential function and a and b are model parameters. The start and fitted values for (a,b) are given by (−0.1,−0.1) and (−0.60,−0.12), respectively. This model captures a gradual decay in P(B).

*Logistic decay:* P(B) = 1/[1 + exp(−a−bx)], where a and b are model parameters. The start and fitted values for (a,b) are given by (0.1,−0.01) and (0.18,−0.21), respectively. This model is equivalent to the logistic regression GLM we fit to the experimental data, but the effect of all explanatory variables apart from cost level has been removed. Under logistic decay, P(B) has a point of inflection at x = −a/b which could be interpreted as a threshold response. However, the function always includes a transition from P(B) = 1 to P(B) = 0 – values we did not observe in the experimental data. To make the identification of a threshold response less ambiguous, we therefore consider a model we call ‘adjusted logistic decay’.

*Adjusted logistic decay:* P(B) = a + (b − a) {(6 − x)^c^/[d^c^ + (6 − x)^c^]}, where a,b,c, and d are model parameters. The start and fitted values for (a,b,c,d) are given by (0.2,0.5,2,3) and (0.27,0.61,2.33,3.56), respectively. This model is explained in more detail in elsewhere^45^. Briefly, under this model P(B) takes values between the minimum a and the maximum b and has a point of inflection for c > 1. As c increases, the function approaches a step-like switch at a threshold of x = d. A model fit with a large value of c and a value of d in the interval [0,6] therefore suggests a threshold response as outlined in hypothesis H2c. We also computed the AIC values for the four models separately for P(B|A) and P(B|notA). *P(B|A).* AIC values for linear decay: 317.46; exponential decay: 317.53; logistic decay: 317.44; adjusted logistic decay: 319.73). *P(B|notA).* AIC values for linear decay: 397.03; exponential decay: 398.02; logistic decay: 397.26; adjusted logistic decay: 400.43).

## Additional Information

**How to cite this article**: Bode, N. W. F. *et al.* Increased costs reduce reciprocal helping behaviour of humans in a virtual evacuation experiment. *Sci. Rep.* **5**, 15896; doi: 10.1038/srep15896 (2015).

## Supplementary Material

Supplementary Information

Supplementary Video S1

Supplementary Video S2

## Figures and Tables

**Table 1:** Overview of summary statistics extracted from data.

Short name	Description	Use
cost	Experimental treatment in which the length of the detour participants have to make to help is changed.	Continuous explanatory variable
event A	A occurs if participants open the door for the other pedestrian from inside the central room.	Response and categorical explanatory variable
event B	B occurs if the computer-controlled pedestrian is rescued.	Response
event C	C occurs if participants open the door for the computer-controlled pedestrian from inside the corridor.	Not used in statistical analysis
age	Participants’ age in years.	Continuous explanatory variable
gender	Participants’ gender (male or female, as there was not enough data on additional categories).	Categorical explanatory variable
training 1	Time taken to move to the first target (log-transformed).	Continuous explanatory variable
training 2	Time taken to return from the first target to the starting position (log-transformed).	Continuous explanatory variable

**Table 2:** Testing the effect of participant characteristics and behaviour on the proportion of participants to help at the first opportunity, P(A).

Effect	Estimate	s.e.	z value	p value
intercept	2.55	1.25	2.04	4.10 × 10^−2^
cost	−0.03	0.04	−0.59	0.55
gender (male)	0.49	0.18	2.78	5.49 × 10^−3^
age	−0.02	0.01	−2.54	1.10 × 10^−2^
training 1	−0.01	0.12	−0.10	0.92
training 2	−0.43	0.17	−2.53	1.14 × 10^−2^

Binomial GLM (logit link function). Response variable: Boolean indicating whether event A occurred. The table shows the estimate, standard error and statistical test results for the explanatory variables in the model. To give an example for interpreting this table: male participants were more likely to help at the first opportunity (increased P(A)) and this effect was unlikely to have arisen by chance (p = 5.49 × 10^−3^).

**Table 3:** Testing the effect of participant characteristics and behaviour on the proportion of participants who helped at the second opportunity, P(B).

Effect	Estimate	s.e.	z value	p value
intercept	−1.64	1.34	−1.23	0.22
cost	−0.23	0.05	−4.80	1.60 × 10^−6^
gender (male)	0.66	0.19	3.41	6.41 × 10^−4^
age	−0.06	0.01	−6.02	1.76 × 10^−9^
A occurs	0.99	0.19	5.16	2.52 × 10^−7^
training 1	0.11	0.13	0.85	0.40
training 2	0.32	0.18	1.80	7.17 × 10^−2^

Binomial GLM (logit link function). Response variable: Boolean indicating whether event B occurred. The table shows the estimate, standard error and statistical test results for the explanatory variables in the model. To give an example for interpreting this table: participants who helped at the first opportunity (A occurs) were more likely to help at the second opportunity (B occurs) and this effect was unlikely to have arisen by chance (p = 2.52 × 10^−7^).

**Table 4:** Testing the effect of participant characteristics and behaviour on the proportion of participants who helped at the second opportunity, given that they had offered help at the first opportunity, P(B|A).

Effect	Estimate	s.e.	z value	p value
intercept	−2.90	2.14	−1.36	0.18
cost	−0.15	0.07	−2.15	3.14 × 10^−2^
gender (male)	0.74	0.29	2.55	1.08 × 10^−2^
age	−0.04	0.02	−2.39	1.70 × 10^−2^
training 1	0.26	0.20	1.34	0.18
training 2	0.38	0.30	1.29	0.20

Binomial GLM (logit link function). Response variable: Boolean indicating whether event B occurred given that A had occurred. The table shows the estimate, standard error and statistical test results for the explanatory variables in the model.

## References

[b1] Penner L. A., Dovidio J. F., Piliavin J. A. & Schroeder D. A. Prosocial behavior: multilevel perspectives. Annu. Rev. Psychol. 56, 365–392 (2005). 15709940 10.1146/annurev.psych.56.091103.070141

[b2] Perugini M., Conner M. & O’Gorman R. Automatic activation of individual differences: a test of the gatekeeper model in the domain of spontaneous helping. Eur. J. Pers. 25, 465–476 (2011).

[b3] Fehr E. & Fischbacher U. The nature of human altruism. Nature 425, 785–791 (2003). 14574401 10.1038/nature02043

[b4] Piliavin J. A. Altruism and helping: the evolution of a field. Soc. Psychol. Q. 72, 209–225 (2009).

[b5] Warneken F. & Tomasello M. Varieties of altruism in children and chimpanzees. Trends Cogn. Sci. 13, 397–402 (2009). 19716750 10.1016/j.tics.2009.06.008

[b6] Hamilton W. D. The genetical evolution of social behaviour I. J. Theor. Biol. 7, 1–16 (1964). 5875341 10.1016/0022-5193(64)90038-4

[b7] Trivers R. L. The Evolution of Reciprocal Altruism. Q. Rev. Biol. 46, 35–57 (1971).

[b8] Axelrod R. & Hamilton W. The evolution of cooperation. Science 211, 1390–1396 (1981). 7466396 10.1126/science.7466396

[b9] Wilson D. S. & Wilson E. O. Rethinking the theoretical foundation of sociobiology. Q. Rev. Biol. 82, 327–348 (2007). 18217526 10.1086/522809

[b10] O’Gorman R., Sheldon K. M. & Wilson D. S. For the good of the group? Exploring group-level evolutionary adaptations using multilevel selection theory. Group Dyn-Theor. Res. 12, 17–26 (2008).

[b11] Wilson D. S., van Vugt M. & O’Gorman R. Multilevel selection theory and major evolutionary transitions implications for psychological science. Curr. Dir. Psychol. Sci. 17, 6–9 (2008).

[b12] Simpson B. & Willer R. Altruism and indirect reciprocity: The interaction of person and situation in prosocial behavior. Soc. Psychol. Q. 71, 37–52 (2008).

[b13] Daly M. & Wilson M. Evolutionary social-psychology and family homicide. Science 242, 519–524 (1988). 3175672 10.1126/science.3175672

[b14] Latané B. & Nida S. “Ten Years of Research on Group Size and Helping”. Psychol. Bull. 89, 308–24 (1981).

[b15] Hamilton W. D. The genetical evolution of social behaviour II. J. Theor. Biol. 7, 17–52 (1964). 5875340 10.1016/0022-5193(64)90039-6

[b16] Dovidio J. F., Piliavin J. A., Schroeder D. A. & Penner L. A. The social psychology of prosocial behavior (Lawrence Erlbaum Associates, Mahwah, NJ, 2006).

[b17] Isaac R. M. & Walker J. M. Group size effects in public goods provision: the voluntary contributions mechanism. Q. J. Econ. 103, 179–199 (1988).

[b18] Andreoni J. & Miller J. Giving according to GARP: an experimental test of the consistency of preferences for altruism. Econometrica 70, 737–753 (2002).

[b19] Stewart-Williams S. Altruism among kin vs. nonkin: effects of cost of help and reciprocal exchange. Evol. Hum. Behav. 28, 193–198 (2007).

[b20] Drury J., Cocking C. & Reicher S. Everyone for themselves? A comparative study of crowd solidarity among emergency survivors. Br. J. Soc. Psychol. 48, 487–506 (2009). 18789185 10.1348/014466608X357893

[b21] Bode N. W. F. & Codling E. A. Human exit route choice in virtual crowd evacuations. Anim. Behav. 86, 347–358 (2013).

[b22] Bode N. W. F., Kemloh Wagoum A. U. & Codling E. A. Human responses to multiple sources of directional information in virtual crowd evacuations. J. R. Soc. Interface 11, 20130904 (2014). 24258157 10.1098/rsif.2013.0904 PMC3869162

[b23] Bode N. W. F., Kemloh Wagoum A. U. & Codling E. A. Information use by humans during dynamic route choice in virtual crowd evacuations. R. Soc. Open Sci. 2, 140410 (2015). 26064589 10.1098/rsos.140410 PMC4448793

[b24] Lipshitz R., Klein G., Orasanu J. & Salas E. Taking stock of naturalistic decision making. J. Behav. Decis. Making 14, 331–352 (2001).

[b25] Gonzalez C., Vanyukov P. & Martin M. K. The use of microworlds to study dynamic decision making. Comput. Hum. Behav. 21, 273–286 (2005).

[b26] Böckler A., Hömke P. & Sebanz N. Invisible man exclusion from shared attention affects gaze behavior and self-reports. Soc. Psychol. Person. Sci. 5, 140–148 (2014).

[b27] Drury J. *et al.* Cooperation versus competition in a mass emergency evacuation: a new laboratory simulation and a new theoretical model. Behav. Res. Methods 41, 957–970 (2009). 19587213 10.3758/BRM.41.3.957

[b28] Levitt S. D. & List J. A. What do laboratory experiments measuring social preferences reveal about the real world? J. Econ Perspect. 21, 153–174 (2007).

[b29] Raihani N. J. & Bshary R. Why humans might help strangers. Front. Behav. Neurosci. 9, 00039 (2015). 10.3389/fnbeh.2015.00039 PMC4335183 25750619

[b30] Leiberg S., Klimecki O. & Singer T. Short-term compassion training increases prosocial behavior in a newly developed prosocial game. PLoS ONE 6, e17798 (2011). 21408020 10.1371/journal.pone.0017798 PMC3052380

[b31] Gigerenzer G. & Gaissmaier W. Heuristic decision making. Annu. Rev. Psychol. 62, 451–82 (2011). 21126183 10.1146/annurev-psych-120709-145346

[b32] Wolf M., Kurvers R. H., Ward A. J., Krause S. & Krause J. Accurate decisions in an uncertain world: collective cognition increases true positives while decreasing false positives. Proc. R. Soc. B 280, 20122777 (2013). 10.1098/rspb.2012.2777 PMC3574371 23407830

[b33] Morimoto C. H. & Mimica M. R. Eye gaze tracking techniques for interactive applications. Comput. Vis. Image Und. 98, 4–24 (2005).

[b34] Iredale W., Van Vugt M. & Dunbar T. Showing off in humans: Male generosity as a mating signal. Evol. Psychol. 6, 386–392 (2008).

[b35] Byrnes J. P., Miller D. C. & Schafer W. D. Gender differences in risk taking: A meta-analysis. Psychol. Bull. 125, 367–383 (1999).

[b36] Baker M. D. Jr & Maner J. K. Risk-taking as a situationally sensitive male mating strategy. Evol. Hum. Behav. 29, 391–395 (2008).

[b37] Lucas K. & Sherry J. L. Sex differences in video game play: a communication-based explanation. Comm. Res. 31, 499–523 (2004).

[b38] Kretz T. *et al.* in Computer Vision Workshops (ICCV Workshops). 2011 IEEE International Conference. 166–172 (IEEE, 2011).

[b39] Proulx G. in SFPE Handbook of Fire Protection Engineering (ed. DiNenno P. J. ) 343–366 (National Fire Protection Association 3rd edition, Quincy, MA, 2002).

[b40] Schadschneider A. *et al.* in Encyclopedia of Complexity and Systems Science (ed. Meyers R. A. ) 3142–3176 (Springer, 2009).

[b41] Braun A., Musse S. R., De Oliveira L. P. L. & Bodmann B. E. J. in Proceedings of the 16th International Conference in Computer Animation and Social Agents (ed. CASA 2003) 143–148 (IEEE, 2003).

[b42] Burstedde C., Klauck K., Schadschneider A. & Zittartz J. Simulation of pedestrian dynamics using a two-dimensional cellular automaton. Physica A 295, 507–525 (2001).

[b43] Helbing D., Farkas I. & Vicsek T. Simulating dynamical features of escape panic. Nature 407, 487–490 (2000). 11028994 10.1038/35035023

[b44] R. Core Team R Foundation for Statistical Computing, Vienna, Austria, *R: a language and environment for statistical computing.* (2013). Available at: http://www.R-project.org/(Accessed: 4th December 2014).

[b45] Sumpter D. J. & Pratt S. C. Quorum responses and consensus decision making. Phil. Trans. R. Soc. B 364, 743–753 (2009). 19073480 10.1098/rstb.2008.0204 PMC2689713

