# Supplementary material for: Increased costs reduce reciprocal helping behaviour of humans in a virtual evacuation experiment
Source: Sci Rep. 2015 Nov 6;5:15896. doi: 10.1038/srep15896 (PMC4635339; doi:10.1038/srep15896)
Supplement: Supplementary Information [file srep15896-s1.pdf]

Supplementary information for

Increased costs reduce reciprocal helping behaviour of humans in a virtual evacuation experiment

Nikolai W. F. Bode[a,b], Jordan Miller[b], Rick O'Gorman[c], Edward A. Codling[b]

[a] Department of Engineering Mathematics, University of Bristol, Bristol, BS8 1UB, UK

[b] Department of Mathematical Sciences, University of Essex, Colchester, CO4 3SQ, UK

[c] Department of Psychology, University of Essex, Colchester, CO4 3SQ, UK

Video legends

Supplementary video S1: video shows one complete experimental run for cost level 0 (square location 1 in figure 1 in the main text). All instruction windows are shown. In the video, the introductory screen is shown for about 21 seconds and we indicate how participants provided information on their gender and age. In the experiment, the introductory screen was shown until participants pressed the start button. The instruction window that initiates the evacuation and therefore the test phase of the experiment includes a green rectangle. In the experiment we showed a commonly used emergency exit sign[1] instead of this green rectangle, which we omit here to avoid licensing issues. The participant-controlled pedestrian is shown in grey. Movements of the mouse cursor are not shown, but two concentric red circles indicate where the participant clicks in the virtual environment to steer the pedestrian. In this example, we show the situation where the participant does not help at the first opportunity (i.e. does not move into the coloured square inside the central room), but does help at the second opportunity (i.e. the computer-controlled pedestrian is allowed to exit the central room). In the video, we do not show experimental data, as the participant-controlled pedestrian is controlled by the lead author.

Supplementary video S2: similar to supplementary video S1, but we show cost level 6 (square location 7 in figure 1 in the main text). In this example, we show the situation where the participant attempts to help at the first opportunity (whilst still inside the central room), but does not help at the second helping opportunity.

Supplementary reference

1. Bundesanstalt für Arbeitsschutz und Arbeitsmedizin, Ausschuss für Arbeitsstaetten, *Technische Regel für Arbeitsstätten ASR A1.3: Sicherheits- und Gesundheitsschutzkennzeichnung* (2013). Available at: www.baua.de/de/Themen-von-A-Z/Arbeitsstaetten/ASR/ASR-A1-3.html (Accessed: 2nd July 2015).
